# Cellular localization of CIP2A determines its prognostic impact in superficial spreading and nodular melanoma

**DOI:** 10.1002/cam4.425

**Published:** 2015-02-07

**Authors:** Vivi Ann Flørenes, Elisabeth Emilsen, Hiep Phuc Dong, Mette Førsund, Ruth Holm, Ana Slipicevic

**Affiliations:** Department of Pathology, Norwegian Radium Hospital, Oslo University HospitalN-0310, Oslo, Norway

**Keywords:** Apoptosis, CIP2A, melanoma, survival, therapy

## Abstract

Cancerous inhibitor of protein phosphatase 2A (CIP2A) is an important oncogene contributing to cancer progression partially by regulating cMYC and AKT. We examined CIP2A expression in cutaneous melanomas, its association with clinicopathological parameters and mapped molecular mechanisms regulated by CIP2A in vitro. CIP2A expression was analyzed by immunohistochemistry in 17 nevi, 132 primary melanomas and 49 metastases. Effects of siRNA-mediated down-regulation on proliferation, apoptosis and signaling pathways were assessed in melanoma cell lines. In superficial spreading melanomas (SSM), high nuclear CIP2A expression was associated with poor overall survival (OS) (*P* = 0.0018). Surprisingly, high cytoplasmic expression was related to improved relapse-free (*P* = 0.031) and OS (*P* = 0.014) in nodular melanomas (NM). In vitro experiments revealed that CIP2A can regulate proliferation and/or apoptosis partially through the PI3K/AKT pathway but also independently. In summary, CIP2A could represent a potential therapeutic target in SSM. However, in NM cytoplasmic CIP2A is associated with improved prognosis indicating that CIP2A has distinct, complex functions dependent on the molecular context and histological subtype. As seen in other cancer types, CIP2A can influence cMYC and AKT, but our data also suggest that in melanoma it has additional targets which need to be identified.

## Introduction

If diagnosed early, cutaneous melanoma can be cured by surgical resection. Unfortunately, however, this tumor type often shows aggressive clinical behavior with high tendency for deadly metastasis and few treatment options. Recently, therapeutic improvements have, however, been achieved by targeting activating BRAF mutation, present in ∽50% of the patients and through immunomodulatory approaches. Still, these promising therapies show limitations such as lack of efficiency in BRAF wild-type patients, fast emergence of resistance and limited and short-lasting responses to immunotherapy 1. Thus, improvement of current treatment strategies and identification of better diagnostic and prognostic biomarkers are still highly warranted.

Cancerous inhibitor of protein phosphatase 2A (CIP2A) is an oncogene shown to promote tumorigenesis and progression of several cancer types. Binding of CIP2A to protein phosphatase 2A (PP2A) complex leads to PP2A inhibition which consequently increases MYC serine 62 phosphorylation and thereby MYC stability, enhancing its transcriptional and oncogenic activities 2,3. Furthermore, the PP2A complex can also dephosphorylate threonine 308 and serine 473 of AKT in a context-dependent manner 4.

Inhibition of CIP2A has been shown to decrease cancer cell viability, anchorage-independent growth and induce apoptosis 5,6. Recently, CIP2A has been reported to regulate cell cycle progression by modulation of polo-like kinase 1 (PLK1) function 7. Consistent with its oncogenic role, overexpression of CIP2A has been found in several tumor types, including ovarian, colon and non–small cell lung carcinoma, as well as in chronic myelogenous leukemia, where it is associated with disease progression and prognostic variables 5,8–15. Moreover, CIP2A has been shown to influence response to therapy in breast and ovarian cancers 9,16,17. To this end and to the best of our knowledge only one study has addressed the possible role of CIP2A in melanocytic lesions, reporting on 18. However, its role in different melanoma subtypes and involved molecular mechanisms have not been investigated. For this reason we have examined CIP2A protein expression in a panel of superficial spreading (SSM) and nodular melanomas (NM) and assessed the relationship to clinical outcome. Using established melanoma cell lines, we have examined whether CIP2A is involved in regulation of major proliferation and survival signaling pathways.

## Material and Methods

### Clinical melanoma specimens

Formalin-fixed, paraffin-embedded tissue from 17 benign nevi, 132 primary melanomas and 49 metastases was examined for expression of CIP2A protein. Of the primaries, 81 were classified as SSM and 51 as NM. Clinical follow-up was available for all patients. Representation in the cohort is 63 male and 69 female, mean age 55.3 (range 19–97). The follow-up period ranged from 1 to 361 months (mean = 105 months, median = 130 months). Cumulative 5 year overall and relapse free survival (RFS) for the total cohort is 71% (73% for SSM and 56% for NM) and 66% (73% for SSM and 53% for NM), respectively. The study was approved by the Regional Committee for medical Research Ethics in Norway.

### Immunohistochemical analysis

Three-*μ*m sections made from formalin-fixed paraffin-embedded tissues were immunostained using the Dako EnVision™+ system (K8012; Dako Cooperation, Carpinteria, CA) and DAKO Autostainer. Deparaffinization, rehydration, and target retrieval were performed in a Dako PT-link and EnVision™ FLEX Target Retrieval Solution, Low pH (citrate buffer, pH 6.1). Endogenous peroxidase was blocked using Dako blocking reagent for 5 min followed by incubation with rabbit polyclonal CIP2A antibody (NB100-74663; Novus Biologicals, Litleton, CO) diluted 1:200 (1.25 *μ*g IgG/mL) for 30 min. Thereafter, the sections were incubated with Dako EnVision™ FLEX+ rabbit linker for 15 min followed by incubation with Dako EnVision™ FLEX/HRP for an additional 30 min. For visualization of staining, the sections were treated with 3′3-diaminobenzidine tetra-hydrochloride (DAB), counterstained with hematoxylin and mounted in Richard-Allan Scientific™ Cytoseal™ XYL (Thermo Scientific, Waltham*,* MA). Negative controls included substitution of polyclonal primary antibody with normal rabbit IgG protein of the same concentration. Four semiquantitative classes were used to describe staining intensity (absent = 0; weak = 1; moderate = 2; strong = 3) and percentage of positive tumor cell (absent = 0; <10% = 1; 10–50% = 2; >50% = 3). Staining in cytoplasm and nucleus were evaluated separately. By multiplying intensity score with percentage positive cell score, a total score index was calculated ranging from 0 to 9. Score index of >2 (cytoplasm) and >0 (nucleus) was considered as high in the statistical analyses.

### Statistical analysis

Statistical analysis was performed applying SPSS package Version 18, (SPSS inc., Chicago, IL) Comparison between variables was performed using the *χ*^2^ test or Fisher exact test. Relationship between CIP2A expression and mean tumor thickness was evaluated using Mann–Whitney two-sample test. Kaplan–Meier survival estimates and log-rank tests were used to evaluate the survival data. Overall survival (OS) is defined as time from primary diagnosis until time of disease related death. RFS is defined as time from primary diagnosis until metastasis, or until the date of the last follow-up. Multivariate survival analysis was performed using the Cox proportional hazard regression model.

Two-tailed paired Student's *t*-test was used for evaluation of in vitro results. A *P*-value of less than 0.05 was considered statistically significant.

### Cell culture

The Wistar Melanoma (WM) cell lines were kindly provided by Dr. Meenhard Herlyn (Wistar Institute, Philadelphia, PA) and have been described elsewhere 19. FEMX-1 cell line was established from a patient treated for melanoma at the Norwegian Radiumhospital, Oslo University Hospital 20. Normal human melanocytes were isolated and cultured as previously described 21. All cells were cultured in RPMI 1640 medium (Bio Whittaker, Verviers, Belgium) supplemented with 5% fetal bovine serum (FBS, PAA Laboratories, Linz, Austria) and 2 mmol/L l-glutamine (GibcoBRL, Paisley, UK) and maintained at 37°C in a humidified 5% CO_2_ atmosphere. The cells were routinely checked for mycoplasma infections and authenticated using the Powerplex 16 kit (Promega, Madison, WI). For inhibition of the PI3K/AKT signaling the cells were treated with 25 *μ*mol/L PI3K inhibitor LY294002 (Cell Signaling Technology Inc, Beverly, MA).

### siRNA knockdown

Cells were transfected with 20 nmol/L siRNA targeting CIP2A (cat no. S100128170; Qiagen, Hilden, Germany) or scrambled control (cat no. 1027280, Qiagen) using Lipofectamine™ RNAiMAX (Invitrogen, Carlsbad, CA) transfection reagent. Cells were harvested for protein and RNA analyses after 48 h, for flow analyses after 72 h and for cell viability assays after 72 and 120 h.

### Quantitative real time RT-PCR

Total RNA was extracted using PerfectPure RNA Cultured Cell Kit (5 Prime Inc., Gaithersburg, MD) according to the manufacturer's description and reverse transcribed with SuperScript® VILO™ cDNA Synthesis Kit (Applied Biosystems, Foster City, CA) using random primers. Real-time RT-PCR analyses were performed as previously described 22 with TaqMan Gene Expression Assay (HS00405413_m1 CIP2A; Applied Biosystems). Relative CIP2A mRNA expression levels were normalized against housekeeping gene beta-glucuronidase (GUSB) (HS99999908_m1 Applied Biosystems). Each sample was run in triplicate. Experiment was performed with two biological replicates.

### Cell viability assay

Effect of CIP2A knockdown on cell viability was assessed after 72 and 120 h using CellTiter 96 Aqueous One solution (MTS assay), as recommended by the manufacturer (Promega, Madison, WI). Absorbance was measured at 490 nm using a microplate Thermo Scientific Multiskan FC reader (Thermo Fisher Scientific, Inc. Waltham, MA). Viability of treated cells was normalized to scrambled siRNA control cells. Each experiment was performed with six parallel observations and at least three biological replicates.

### Immunoblotting

Immunoblotting was performed as previously described 21. All membranes were blocked for 1 h in 5% nonfat milk in TBS buffer supplemented with 0.1% Tween and probed at 4°C over night with Caspase 3 (#AF-605-NA dilution 1:2000), PARP (#9532; 1:2000), p38 (#9212; 1:1000), p-p38 (#4631; 1:1000), JNK (#9258; 1:2000), p-JNK (#9255; 1:2000), AKT (#9272 1:1500) and pAKT (#9271 1:1000) from Cell Signaling Technology, Inc.; pMAPK (#V8031; 1:1000) Promega; and *α*-tubulin (#CP06; 1:5000) Calbiochem. Secondary HRP-conjugated anti-rabbit or anti-mouse IgG antibodies were from Promega. Visualization was performed using ECL-plus chemifluorescent reagent (GE Healthcare, Chalfont St. Giles, UK). Densiometric analysis was performed using ImageJ software (Rasband, W.S., ImageJ, U. S. National Institutes of Health, Bethesda, MD, http://imagej.nih.gov/ij/, 1997–2012.) where all lanes were normalized against their respective *α*-tubulin loading controls.

### Flow cytometric cell cycle analysis

Fixed cells were washed 1× in PBS and stained with premade commercial propidium iodide/RNAse solution (Cytognos S.L, Salamanca, Spain). Flow cytometric analysis was performed using FACS Calibur flow cytometer equipped with a 15-mW argon-ion laser (488 nm) and 12-mW red diode laser (635) (BD Biosciences, San Jose, CA) and results analyzed with FlowJo analysis software (Version 9.7.5; Tree Star, Ashland, OR).

## Results

### Expression of CIP2A in melanocytic lesions

**Figure 1:**
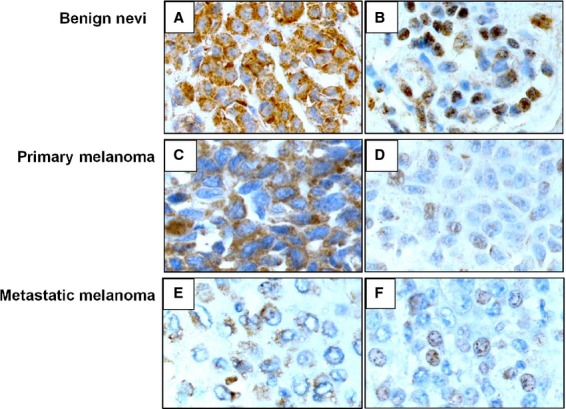
Immunohistochemical staining of cancerous inhibitor of protein phosphatase 2A in cytoplasm and nucleus of benign nevus (A and B), primary melanoma and (C and D) and metastatic melanoma (E and F).

Expression of CIP2A protein was analyzed by immunohistochemistry in a panel of paraffin-embedded benign nevi, primary and metastatic melanomas. As illustrated in Figure 1 cytoplasmic and/or nuclear expression was observed. High cytoplasmic expression (score index >2) was found in 89 (67%) of the primaries whereas 3 (10%) had weak (score index ≤2) and 30 (23%) lacked immunoreactivity (Tables 1 and S1). Twenty-six cases (20%) had high nuclear CIP2A expression (score index >0) whereas the remaining primary tumors (106) displayed no nuclear reactivity. There were no notable differences with regard to score index between SSM and NM. Thirty-five (72%) metastatic tumors had high cytoplasmic staining score index, 5 (10%) weak and 9 (18%) were negative. Seventeen (35%) of the metastases also expressed high nuclear CIP2A. Cytoplasmic reactivity was seen in all 17 nevi examined of which 12 (71%) showed high expression. In contrast to the tumor specimens, most nevi (94%) had high nuclear staining.

**Table 1:** Expression of CIP2A according to subcellular localization

	No. cases (%) with high^1^ score index	
	Cytoplasm	Nucleus
Nevi	12 (71)	16 (94)
Melanoma	89 (67)	26 (19)
SSM	55 (68)	13 (16)
NM	34 (67)	13 (26)
Metastasis	35 (72)	17 (35)

CIP2A, cancerous inhibitor of protein phosphatase 2A; SSM, superficial spreading melanoma; NM, nodular melanoma.

1 High score index defined as >2 in cytoplasm and >0 in nucleus.

### Expression of CIP2A in relation to clinical parameter and disease outcome

**Figure 2:**
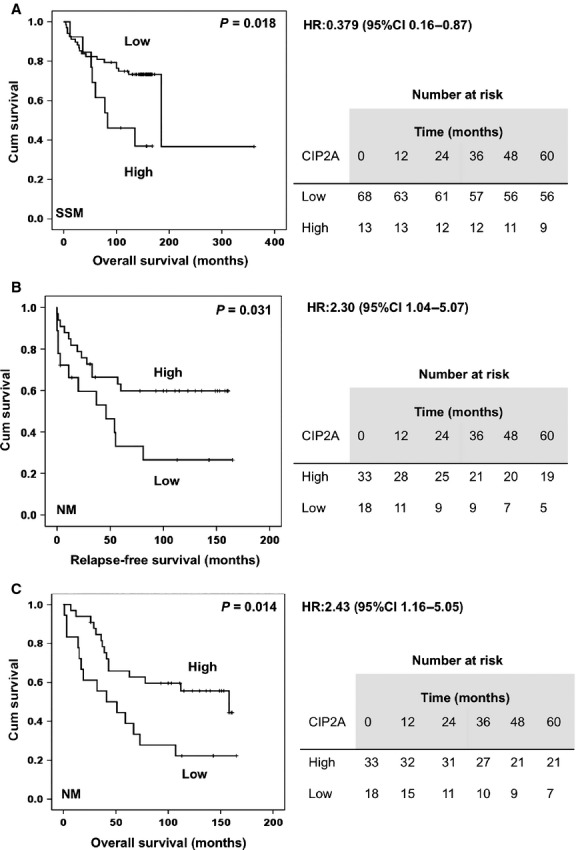
(A) Kaplan–Meier survival curve showing the impact of high nuclear CIP2A expression and OS in superficial spreading melanoma (*n* = 81). (B) Kaplan–Meier survival curve showing impact of high cytoplasmic CIP2A expression and RFS and (C) OS survival in nodular melanoma (*n* = 51). Tables next to respective curves show number of patients at risk. CIP2A, cancerous inhibitor of protein phosphatase 2A; OS, overall survival; RFS, relapse-free survival.

Analysis of cytoplasmic CIP2A expression in SSM did not reveal any impact on RFS and OS whereas high nuclear expression (score index >0) indicated poor OS (*P* = 0.018 Log-rank test) (Fig. 2A). The cumulative 5-year survival rate was 81.1% (mean survival time 212.1 months; 95% CI: 119.3–304.9) in the low CIP2A expression group, whereas it was 69% (mean survival time 103.3 months; 95% CI: 72.3–134.3) in the high CIP2A expression group. CIP2A expression, in addition to previously identified melanoma prognostic factors including tumor depth, ulceration, Cyclin A and Ki67 expression, were analyzed using univariate and multivariate Cox regression analysis (Table S2). Only tumor depth and Ki67 remained independent prognostic factors (*P* < 0.05) while following the analysis.

Surprisingly, high cytoplasmic CIP2A expression (score index >2) was indicative of improved RFS (*P* = 0.031 Log-rank test) and OS (*P* = 0.014 Log-rank test) in NM (Fig. 2B and C). The cumulative 5-year RFS was 63% (mean survival time 105.3 months; 95% CI: 81.6–129.1) in the high CIP2A expression group whereas it was only 33% (mean survival time 64 months; 95% CI: 32.4–95.6) in the low CIP2A expression group. Cumulative 5 years OS was 66% (mean survival time 108.7 months; 95% CI: 87.7–129.7) in the high CIP2A expression group and 39% (mean survival time 64.5 months; 95% CI: 36.7–92.3) in the low CIP2A expression group. Furthermore, impact on OS was further increased (*P* = 0.005 Log-rank test) if all cytoplasmic immunoreactivity (score index >0) was defined as high (data not shown). In addition, CIP2A cytoplasmic expression remained an independent prognostic factor (*P* = 0.044) in this patient group in the multivariate analysis (Table S2). Nuclear reactivity showed no association to these parameters. Neither cytoplasmic nor nuclear CIP2A immunoreactivity was associated with tumor thickness and ulceration in any of the subtypes (data not shown). Furthermore, there was no impact of CIP2A expression on OS in patients with metastatic disease.

As our panel of melanomas has previously been analyzed for regulators of the cell cycle (Ki-67, Cyclin A, D1, D3, p21^CIP1/WAF1^, p27^Kip1^ and p53) 23–27 and activation of signaling pathways (AKT, MAPK, p38, JNK) 28,29 we examined the relationship between CIP2A and these factors. A negative correlation between high cytoplasmic CIP2A and p53 (*P* = 0.011 Fisher's exact test) and a negative trend with nuclear p-AKT (*P* = 0.059 *χ*^2^) was found in SSM (Table 2). However, none of the markers were associated with nuclear or cytoplasmic CIP2A expression in NM but if all cytoplasmic CIP2A immunoreactivity was defined as high (score index >0) a negative correlation (*P* = 0.016 Fisher's exact test) between CIP2A and AKT was observed.

**Table 2:** Associations between clinical parameters, signaling pathways, and CIP2A expression

	CIP2A				
Clinical parameter	No. analyzed	Expression	Low	High	*P*-value
SSM					
Mean tumor depth	78		2.06 mm	1.68 mm	0.538^1^
Marker					
P53	49	Low^2^	13	32	0.011^3^
		High	4	0	
Nuclear pAkt	75	Low^4^	15	44	0.059
		High	8	8	
NM					
Mean tumor depth	50		5.09 mm	4.6 mm	0.395^1^
Marker					
Cytoplasmic pAkt	45	Low^5^	0	19	0.016^3^
		High	7	19	

CIP2A, cancerous inhibitor of protein phosphatase 2A; SSM, superficial spreading melanoma; NM, nodular melanoma.

1 Obtained by Mann–Whitney test.

2 Low expression defined as immunoreactivity in <5% of tumor cells.

3 Obtained by Fisher's exact test.

4 Low expression defined as immunoreactivity in <5% of tumor cells.

5 Low expression defined as immunoreactivity in 50% or less of tumor cells.

### CIP2A protein levels are increased in melanoma cell lines

**Figure 3:**
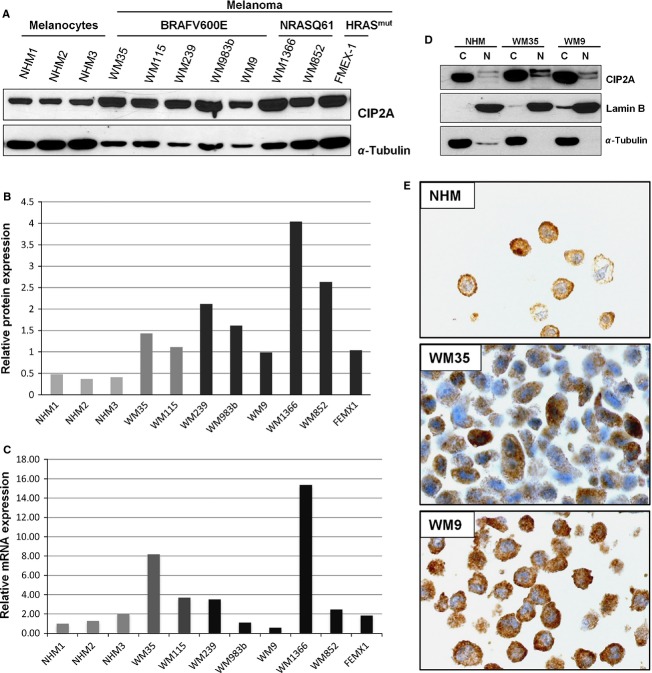
Characterization of CIP2A expression in a panel of normal human melanocytes (NHM) and melanoma cell lines. (A) Immunoblot analysis and (B) quantification of protein expression levels of CIP2A (C) CIP2A mRNA expression measured by qRT-PCR. NHM1 line was used as calibrator. (D) Cytoplasmic and nuclear expression of CIP2A in normal melanocytes, primary and metastatic melanoma cell lines assessed by immunoblot analysis and (E) by immunohistochemical staining. CIP2A, cancerous inhibitor of protein phosphatase 2A.

Next we assessed the levels of CIP2A mRNA and protein in a panel of normal human melanocytes (NHM) and established melanoma lines. As shown in Figure 3A and B, immuno-blot analysis revealed that melanoma cell lines had an increased level of CIP2A protein compared to cultured melanocytes. Increased CIP2A protein levels were partially correlated to increased mRNA levels (Fig. 3C), suggesting that both pre- and posttranscriptional regulation might occur. There was no obvious difference between the cell lines in relation to their genetic background including BRAF^V600E^ and NRAS^Q61K^, PTEN loss or HRAS mutations. Furthermore, assessment of the cytoplasmic and nuclear cell fractions showed that CIP2A is predominately found in the cytoplasm of cultured cells including melanocytes. Interestingly, a week nuclear staining was also detected in all lines by both immunoblotting and immunohistochemistry (Fig. 3D and E).

### CIP2A regulates proliferation and/or apoptosis depending on genetic context of the cells

**Figure 4:**
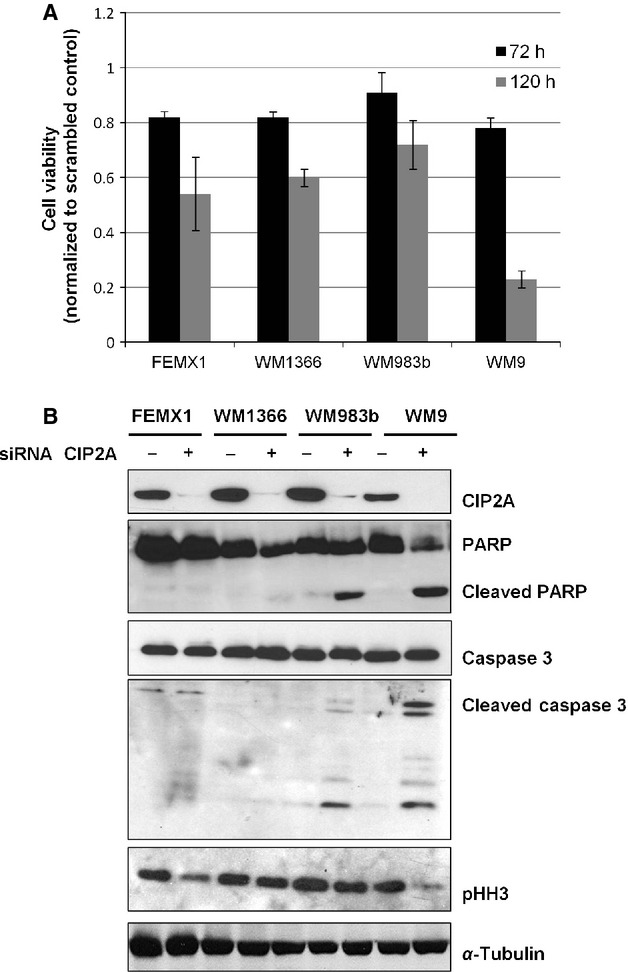
CIP2A downregulation reduces proliferation and induces apoptosis in melanoma cells. (A) The relative amount of viable cells measured by MTS assay 72 and 120 h post siRNA transfection. (B) Immunoblot analysis showing reduction in cancerous inhibitor of protein phosphatase 2A protein levels 48 h post siRNA transfection and effects on proliferation and apoptosis markers. The figure is representative of at last three independent biological experiments.

To further elucidate the role of CIP2A we transiently downregulated the protein in four melanoma cell lines harboring the common genetic mutations *BRAF*^*V600E*^ (WM983b), *BRAF*^*V600E*^*/PTEN*^*null*^ (WM9), *NRAS*^*Q61K*^ (1366) and the less frequently seen *HRAS* mutation (FEMX1). This downregulation was accompanied with ∽20% reduction in cell viability (Figs.4A and S1). However, prolonged incubation for up to 120 h led to further decrease in viability. The effect was most profound in WM9 cells showing ∽80% inhibition. In order to clarify whether the observed decrease in viability was attributed to decreased proliferation and/or apoptosis we analyzed the expression of the mitosis marker phospho-Histone H3 (*pHH3*) and cleavage status of the apoptotic markers Poly (ADP-ribose) polymerase (PARP) and Caspase 3 by immunoblotting. As shown in Figure 4B in the *BRAF*^*V600E*^ mutant WM983b and WM9 cell lines CIP2A downregulation led to cleavage of PARP and Caspase 3 indicating induction of apoptosis whereas this was not the case in *NRAS*^*Q61K*^ mutant WM1366 and *HRAS* mutant FEMX1 cells. In the latter cell lines reduced expression of pHH3 and a minor accumulation of cells in G1 phase (data not shown) supportive of growth arrest were observed.

**Figure 5:**
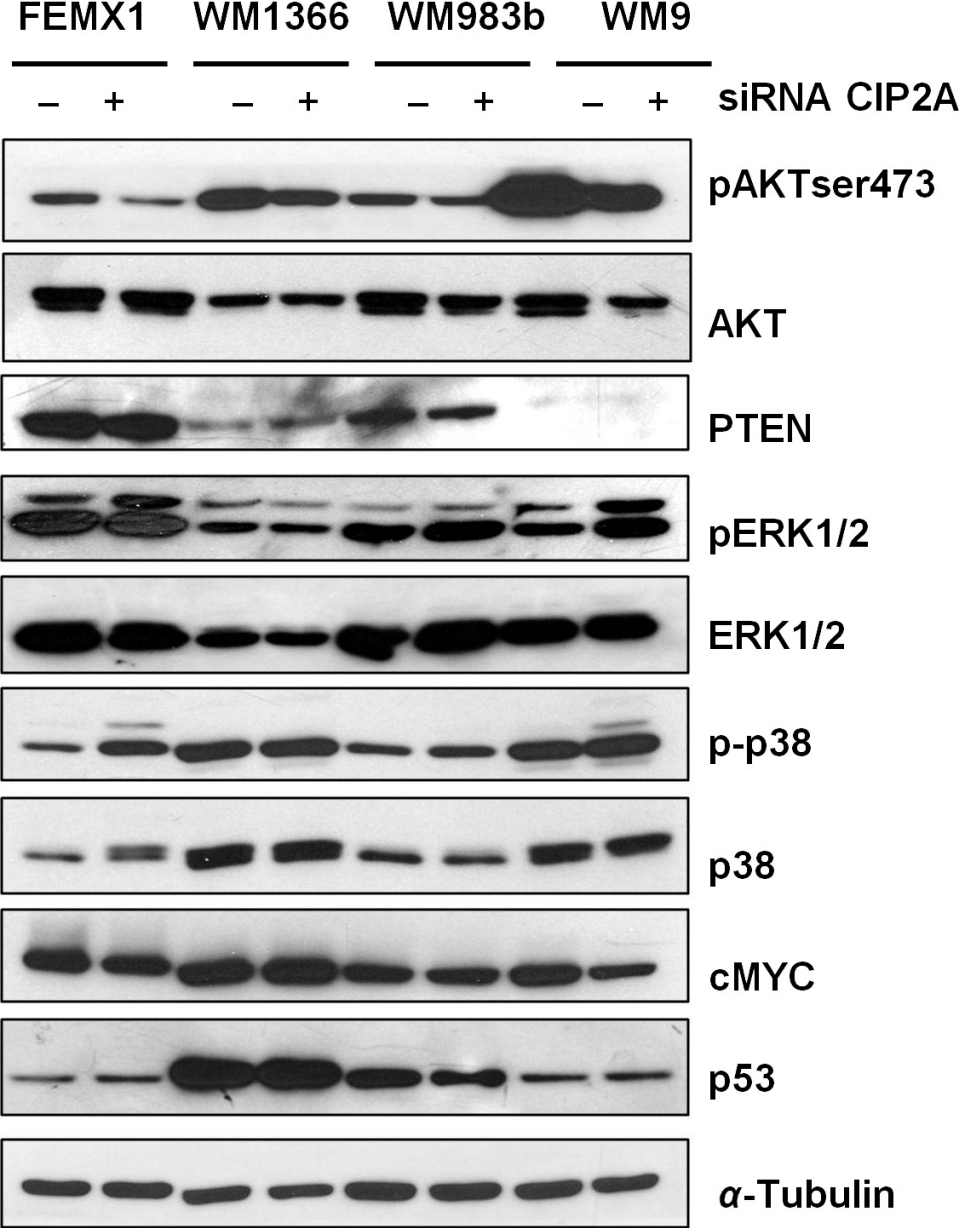
Effects of cancerous inhibitor of protein phosphatase 2A downregulation on cell signaling 48 h after siRNA transfection. The figure is representative of at last three independent biological experiments.

To examine which signaling pathways might mediate these effects we next examined activation status of the PI3K/AKT and MAPK kinase pathways (MAPK\ERK, JNK, p38MAPK) and protein levels of cMYC and p53 48 h after CIP2A downregulation. As shown in Figure 5 we observed a slight decrease in Ser473 AKT phosphorylation in all cell lines whereas there was little or no effect on the MAPK pathways. The level of p53 was not notably affected in any of the lines whereas only a weak downregulation of cMYC was observed in WM9 cells.

The most profound phenotypic effects of CIP2A downregulation were observed in *BRAF*^*V600E*^*/PTEN*^*null*^ WM9 cells, expressing the highest level of activated AKT. For this reason we wanted to test if these effects are dependent on AKT. In such case, it could be expected that inhibition of the PI3K/AKT pathway using PI3K inhibitor LY294002 would yield similar results. As shown in Figure S2, 48 h treatment with 25 *μ*mol/L LY294002 led to a more efficient reduction of pAKT and cMYC levels than CIP2A knockdown. However, in contrast to CIP2A knockdown, LY294002 treatment did not induce cleavage of Caspase 3, suggesting that CIP2A might regulate apoptosis independently of PI3K/AKT pathway in melanoma cells. In addition, we did not observe any effects on the level of pS6 protein suggesting that mammalian target of rapamycin (mTOR) activity is not significantly affected by CIP2A downregulation.

## Discussion

In this study we have examined expression, clinical relevance and function of CIP2A in melanoma tissues, primary melanocyte cultures and established cell lines. We found CIP2A to be highly expressed in both nevi and melanomas, showing both cytoplasmic and nuclear staining. Interestingly, while cytoplasmic CIP2A expression was comparable between the lesions, high nuclear expression was observed more frequently in nevi. Several previous studies have demonstrated overexpression of CIP2A in various tumor types. However, to best of our knowledge, only one previous study has reported on primarily cytoplasmic CIP2A expression in melanomas, which was higher in tumor samples compared to nevi and correlated with poor OS and Breslow thickness 18. In contrast to these results, we found that nuclear CIP2A expression is a marker of reduced OS in SSM whereas this is not the case for NM, suggesting that CIP2A has distinct functions in different melanoma subtypes. As the former study did not specify which antibody that was used, we cannot exclude that this discrepancies might be due to the use of different antibodies. In addition, the melanoma subtypes were not specified. In support of our results, Bockelman et al. observed CIP2A nuclear staining in ovarian cancer tissue and cell lines using the same antibody as in this study 9. Interestingly, however, they found that high nuclear expression was associated with prolonged OS suggesting that CIP2A functions are also tumor type specific. Two recent studies provide support for a nuclear role of CIP2A, first demonstrating that cytoplasmic CIP2A can shuttle into the nucleus when nuclear export factor is inhibited and second showing that CIP2A is enriched in the nucleus of human cancer cells during entry into mitosis 7,30. For this reason it is not unlikely that nuclear CIP2A might be important for mitotic progression of SSM, proliferation and thus worse clinical outcome. In light of these results, our observation of high nuclear CIP2A immunoreactivity seen in benign nevi might be difficult to explain at this point. However, we have also observed a weak nuclear expression of CIP2A in normal cultured melanocytes, which further suggests that CIP2A has a context-dependent role in melanoma progression. Furthermore, the majority of benign nevi are senescent lesions that do not progress to melanoma and hence CIP2A might have a different role in this setting. A parallel to our observations is high expression of oncogenic BRAF^V600E^ protein in nevi.

Whereas we did not find any correlation between cytoplasmic CIP2A expression and clinical parameters in SSM, it was surprisingly related to both improved RFS and OS in NM. This is in contrast to previous studies in ovarian, tongue and non–small cell lung cancers where cytoplasmic CIP2A is reported to be an independent marker of reduced OS 9,14,31. However, in studies of breast and colon cancer no such association was observed 5,8. An increasing line of evidence suggests that SSM and NM have distinct molecular pathogenesis and different clinical manifestation and our results are underlining this molecular heterogeneity as well as context-dependent CIP2A functionality 32.

In our study, CIP2A downregulation led either to cell type specific inhibition of proliferation and/or apoptosis. This is in accordance with previous report demonstrating decreased cell viability and anchorage independent growth following CIP2A inhibition in breast cancer cells 5. It has also been shown that CIP2A can protect cancer cells from therapy-induced apoptosis 33. These effects are partially mediated by cMYC and AKT, which are known CIP2A targets. For instance, in liver cancer, CIP2A overexpression increased AKT phosphorylation. Still, these effects are clearly cell type dependent as CIP2A depletion did not affect AKT phosphorylation in various colon cancer cell lines whereas it potently inhibited c-MYC expression 12,34. Our in vitro results show that CIP2A downregulation slightly reduced the pAKT levels in all melanoma cell lines regardless of their genetic background whereas regulation of cMYC was less profound. Interestingly, in patient-derived samples we observed a negative trend between cytoplasmic CIP2A expression and nuclear pAKT in SSM whereas in NM cytoplasmic CIP2A was negatively associated with cytoplasmic pAKT suggesting that effects on AKT regulation might be dependent on CIP2A cellular localization. Together these results suggest that AKT might be a CIP2A target in melanoma.

Inhibitory effects of CIP2A downregulation on proliferation of melanoma cells were not related to the genetic background of the cells. Some of the effects might be attributed to a slight pAKT and cMYC downregulation Elevated level of cMYC due to PP2A downregulation, has been shown to stimulate proliferation and suppress oncogene-induced senescence in melanocytes and melanoma cells, suggesting a role of cMYC in melanoma progression 35.

CIP2A downregulation led to induction of apoptosis only in *BRAF*^*V600E*^ mutant lines but without affecting the MAPK/ERK kinase pathway. Besides, inhibition of PI3K/AKT and reduction in cMYC levels alone were not sufficient to induce apoptosis, suggesting that CIP2A regulates additional survival factors. Our results are supported by a recent study by Silva et al. showing that AKT inhibitors reduced cell viability with up to 50% in WM9 (*BRAF*^*V600E*^*/PTEN*^*null*^) cells without an increase in apoptosis 36. While it is known that PTEN loss contributes to the intrinsic resistance of *BRAF*^*V600E*^ -mutated cell lines to *BRAF*^*V600E*^ inhibitors, our results show that these cells are highly sensitive to CIP2A downregulation, suggesting that targeting CIP2A might be a good therapeutic alternative.

Recently, it has been shown that in breast cancer cells, CIP2A associates with mammalian target of rapamycin complex 1 (mTORC1) and enhances mTORC1-dependent growth signaling and inhibits autophagy independently of cMYC and AKT 37. Also in melanoma cells mTORC1/2 signaling can regulate proliferation independently of and more potently than AKT 36. For this reason, it is possible that CIP2A might regulate mTORC1 and autophagy in melanoma, which indirectly might affect apoptosis. Crosstalk between autophagy and apoptosis has previously been widely reported 38. Interestingly, upregulation of autophagy-related proteins Beclin 1 and LC3A has been reported in NM and their expression was associated with poor OS 39. Thus, inhibition of autophagy by CIP2A could partially explain the surprising protective role of cytoplasmic CIP2A in NM. However, downregulation of CIP2A did not influence activity of S6 protein, a target of mTORC1/2 in WM9 cell line, suggesting CIP2A might not be involved in regulation of mTORC1/2 pathway in this line.

In conclusion, our results show that nuclear CIP2A is a predictor of poor OS in SSM where it acts as an oncogene and might represent a potential therapeutic target. However, due to its diverse multitarget regulatory potential it has additional, to date not yet determined functional role in NM that require careful further investigation.

## Conflict of Interest

None declared.

## Supporting Information

**Figure S1.** Relative amount of viable cells measured by MTS assay 72 and 120 hours post siRNA transfectio normalized to untreated controls. The figure is representative of at last three independent biological experiments.

**Figure S2.** Immuno-blot analysis of AKT activation and Caspase 3 cleavage in WM9 cells 48 hours after treatment with 25μM PI3K inhibitor LY294002 or transfection with siRNA targeting CIP2A. The figure is representative of at last three independent biological experiments.

**Table S1.** Total score index of CIP2A according to subcellular localization.

**Table S2.** Univariate and multivariate Cox regression analysis of CIP2A expression levels and survival rates.
