# Supplementary figures and images for: Cellular localization of CIP2A determines its prognostic impact in superficial spreading and nodular melanoma
Source: Cancer Med. 2015 Feb 7;4(6):903–13. doi: 10.1002/cam4.425 (PMC4472213; doi:10.1002/cam4.425)

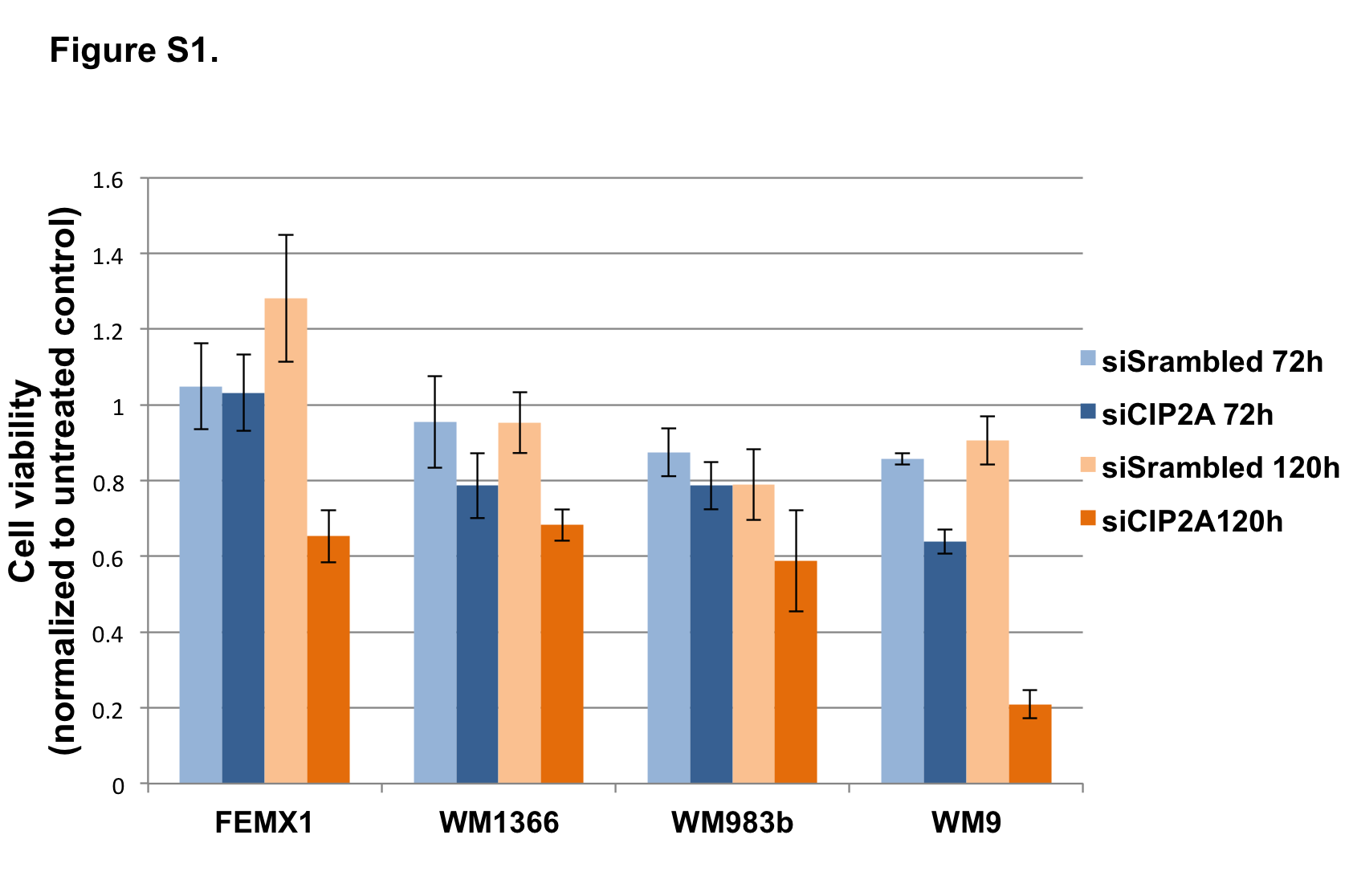

Supplement: Supplementary file 1 [file cam40004-0903-sd1.tif]

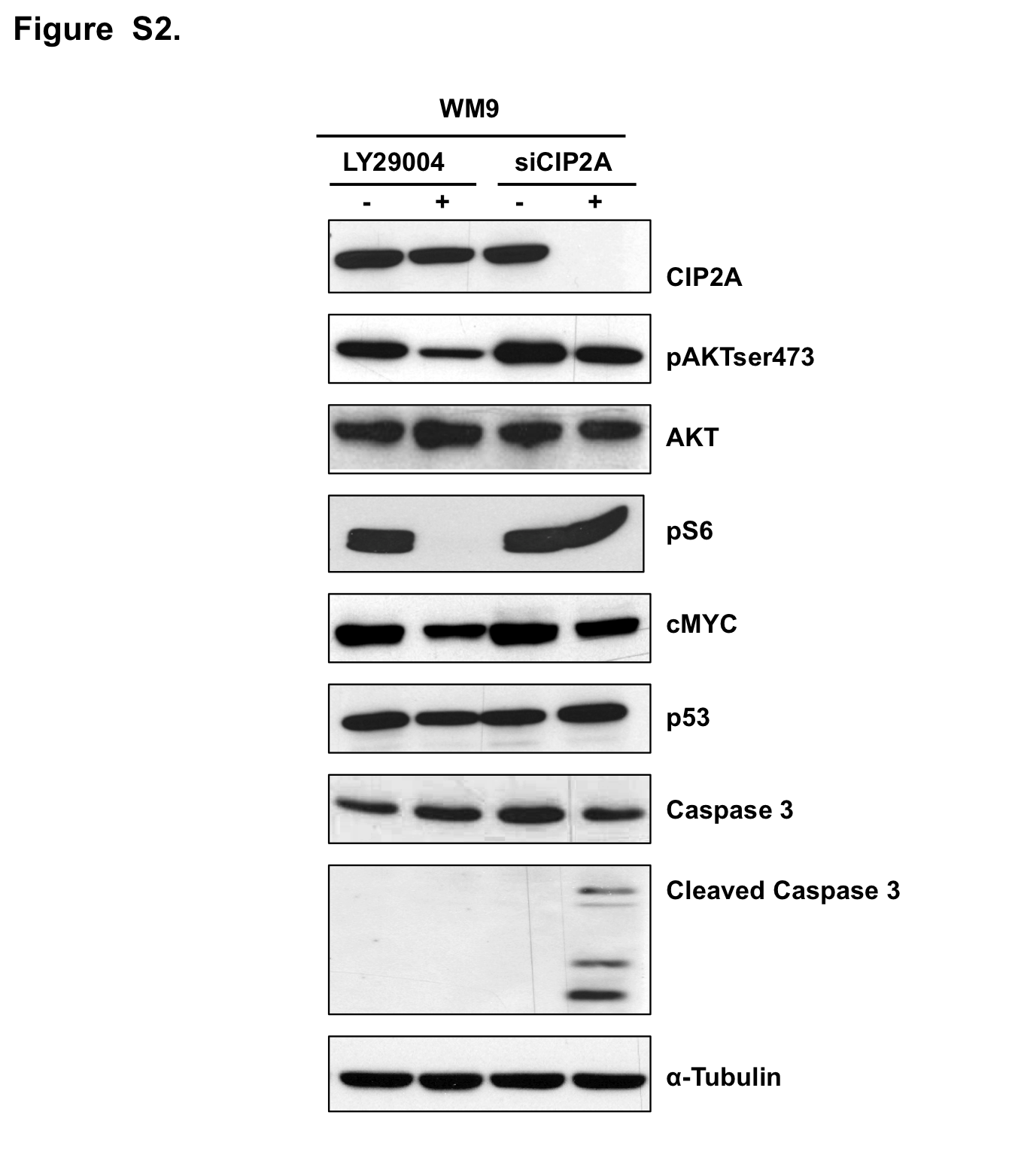

Supplement: Supplementary file 2 [file cam40004-0903-sd2.tif]
