# Supplementary material for: Cellular localization of CIP2A determines its prognostic impact in superficial spreading and nodular melanoma
Source: Cancer Med. 2015 Feb 7;4(6):903–13. doi: 10.1002/cam4.425 (PMC4472213; doi:10.1002/cam4.425)
Supplement: Supplementary file 3 [file cam40004-0903-sd3.docx]

**Supplementary table 1.** Total score index of CIP2A according to subcellular localization

|  | | **Cytoplasm** | | | | | | **Nucleus** | | | | |
| --- | --- | --- | --- | --- | --- | --- | --- | --- | --- | --- | --- | --- |
|  | **Score index**  **No.** | **0** | **2** | **3** | **4** | **6** | **9** | **0** | **2** | **3** | **4** | **6** |
| **Nevi** | **17**  **(%)** | **/** | **5**  **(29)** | **10**  **(59)** | **1**  **(6)** | **/** | **1**  **(6)** | **1**  **(6)** | **6**  **(35)** | **3**  **(18)** | **7**  **(41)** | **/** |
| **Primary** | **132** | **30**  **(23)** | **13**  **(10)** | **36**  **(27)** | **21**  **(16)** | **28**  **(21)** | **4**  **(3)** | **106**  **(80)** | **8**  **(6)** | **14**  **(11)** | **4**  **(3)** | **/** |
| SSM | 81 | 20  (25) | 6  (7) | 22  (27) | 14  (17) | 16  (20) | 3  (4) | 68  (84) | 3  (4) | 8  (10) | 2  (2) | / |
| NM | 51 | 10  (19) | 7  (14) | 14  (27) | 7  (14) | 12  (24) | 1  (2) | 38  (74) | 5  (10) | 6  (12) | 2  (4) | / |
| **Met** | **49** | **9**  **(18)** | **5**  **(10)** | **16**  **(33)** | **7**  **(14)** | **11**  **(23)** | **1**  **(2)** | **32**  **(65)** | **3**  **(6)** | **13**  **(27)** | **/** | **1**  **(2)** |
