# Supplementary material for: Cellular localization of CIP2A determines its prognostic impact in superficial spreading and nodular melanoma
Source: Cancer Med. 2015 Feb 7;4(6):903–13. doi: 10.1002/cam4.425 (PMC4472213; doi:10.1002/cam4.425)
Supplement: Supplementary file 4 [file cam40004-0903-sd4.docx]

**Table S2. Univariate and multivariate Cox regression analysis of CIP2A expression levels and survival rates**

|  |  | **Univariate analysis** | | | **Multivariate analysis** | | |
| --- | --- | --- | --- | --- | --- | --- | --- |
|  |  | **Overall survival** | | | | | |
| **SSM** | **Variable** | **HR** | **95% CI** | ***P-*value** | **HR** | **95% CI** | ***P-*value** |
|  | Tumor Depth | 1.1 | 1,06-1.32 | 0,002 | 1,154 | 1,01-1,31 | 0,026 |
|  | Ki67 | 0.29 | 0.13-0,69 | 0,003 | 0,38 | 0,16-0,86 | 0,021 |
|  | Cyclin A | 0.37 | 0,16-0,82 | 0,015 |  |  |  |
|  | CIP2A^1^ (Low vs High) | 0.37 | 0,16-0,87 | 0,023 |  |  |  |
|  | Ulceration | 0.51 | 0,22-1,18 | 0.119 |  |  |  |
|  |  |  |  |  |  |  |  |
| **NM** |  | **Overall survival** | | | | | |
|  | Tumor Depth | 1.14 | 1.05-1,23 | 0,001 | 1,15 | 1,04-1,27 | 0,011 |
|  | Ki67 | 1.02 | 0.46-2.23 | 0,961 |  |  |  |
|  | Cyclin A | 0,84 | 0,36-1,95 | 0,685 |  |  |  |
|  | CIP2A^2^ (Low vs High) | 2,43 | 1,16-5,0 | 0,018 | 2,54 | 1.0-6.05 | 0,032 |
|  | Ulceration | 0,43 | 0.18-1,23 | 0,071 |  |  |  |
|  |  |  |  |  |  |  |  |
|  |  | **Relapse free survival** | | | | | |
|  | Tumor Depth | 1,21 | 1,1-1,37 | 0,000 | 1,21 | 1,1-1,3 | 0,000 |
|  | Ki67 | 1,26 | 0,55-2,8 | 0,569 |  |  |  |
|  | Cyclin A | 0,98 | 0,41-2,32 | 0,984 |  |  |  |
|  | CIP2A^2^*(Low vs High) | 2,30 | 1,04-5,0 | 0,038 | 2,26 | 1,0-5 | 0,044 |
|  | Ulceration | 0,61 | 0,25-1,4 | 0,280 |  |  |  |

^1^ Nuclear CIP2A

^2^ Cytoplasmic CIP2A
